# Exploring the extrachromosomal plasmid rDNA of *Naegleria fowleri* AY27 genotype II: A human brain-eating amoeba via high-throughput sequencing

**DOI:** 10.1186/s12920-024-01890-y

**Published:** 2024-05-07

**Authors:** Muhammad Aurongzeb, Hafiz Muhammad Talha Malik, Muhammad Jahanzaib, Syed Shah Hassan, Yasmeen Rashid, Tariq Aziz, Metab Alharbi

**Affiliations:** 1 Department of Biotechnology, Faculty of Engineering Sciences & Technology, Hamdard University, Karachi, 74600 Pakistan; 2 Alpha Genomics Private limited, Islamabad, Pakistan; 3 JRC Genome Research, PCMD, International Center for Chemical and Biological Sciences, University of Karachi, Karachi, 75270 Pakistan; 4 Department of Biochemistry, University of Karachi, Karachi, 75270 Pakistan; 5 Laboratory of Animal Health Food Hygiene and Quality, Department of Agriculture, University of Ioannina, Arta, 47132 Greece; 6 Department of Pharmacology and Toxicology College of Pharmacy, King Saud University, Riyadh, Saudi Arabia

**Keywords:** Free-living amoeba, *Naegleria fowleri*, High-throughput sequencing, Primary amebic meningoencephalitis (PAM), Circular extrachromosomal ribosomal DNA element (CERE rDNA), virtual screening and docking

## Abstract

*Naegleria fowleri*, also known as brain-earing amoeba, causes severe and rapidly fatal CNS infection in humans called primary amebic meningoencephalitis (PAM). The DNA from the *N. fowleri* clinical isolate was sequenced for circular extrachromosomal ribosomal DNA (CERE - rDNA). The CERE contains 18 S, 5.8 S, and 28 S ribosomal subunits separated by internal transcribed spacers, 5 open reading frames (ORFs), and mostly repeat elements comprising 7268 bp out of 15,786 bp (46%). A wide variety of variations and recombination events were observed. Finally, the ORFs that comprised only 4 hypothetical proteins were modeled and screened against Zinc drug-like compounds. Two compounds [ZINC77564275 (ethyl 2-(((4-isopropyl-4 H-1,2,4-triazol-3-yl) methyl) (methyl)amino) oxazole-4-carboxylate) and ZINC15022129 (5-(2-methoxyphenoxy)-[2,2’-bipyrimidine]-4,6(1 H,5 H)-dione)] were finalized as potential druggable compounds based on ADME toxicity analysis. We propose that the compounds showing the least toxicity would be potential drug candidates after laboratory experimental validation is performed.

**Supplementary Information:**

The online version contains supplementary material available at 10.1186/s12920-024-01890-y.

## Introduction

*Naegleria fowleri*, also known as brain-eating amoeba, causes severe and rapidly fatal CNS infection in humans called primary amebic meningoencephalitis (PAM). Its habitat is naturally occurring as well as artificial warm freshwater bodies like spas, pools, and domestic water reservoirs. Being a thermophilic organism, *N. fowleri* multiplies quickly at higher temperatures, i.e., 40–46 °C [1]. During the last fifty years, several PAM cases have been recorded from different countries; the reason for this increased prevalence worldwide seems to be global warming [2]. According to a study, Pakistan had the second highest prevalence of Naegleria infections around the world [3]. Mostly, PAM cases have been associated with a recent history of warm freshwater swimming or direct contact with contaminated tap water [4–6]. The intermingling symptomatology of PAM with bacterial meningitis usually results in the late diagnosis of this disease [4, 7]. However, lumbar puncture of suspected patients having severe headache, fever, nausea, and a low Erythrocyte Sedimentation Rate (ESR) should be immediately done [8].

In 2008, the first PAM patient was diagnosed in Karachi, Pakistan. A*n obvious increase in PAM incidences has been reported during the last few years.* Despite the morphological resemblance among *N. fowleri* isolates, eight distinct *N. fowleri* genotypes (Genotype-1 to Genotype-8) have been categorized across the globe based on differences in ribosomal internal transcribed spacers, including 5.8 S rDNA [9]. Additionally, their abundance is quite unequal around the globe; three genotypes, i.e., 1, 2, and 3, have been identified in America, seven genotypes, i.e., 2–8, in Europe, and only two genotypes, i.e., 2 and 3, in Asia. However, only four genotypes, i.e., 1, 2, 3, and 5 have been recognized to be pathogenic [10]. Genotyping studies aim to identify accurate pathogenic genotype so that effective genotype-specific vaccines or drugs can be developed. Moreover, the different geographical distribution of varied genotypes makes them a vital epidemiologic indicator, which can be employed in mapping out the right source of infection in certain populace [11]. *N. fowleri* genotype II has previously been reported from many Asian countries including Pakistan [12, 13].

*N. fowleri* identification and treatment is key to reducing mortality caused by this pathogen. With the advancement in genetics in recent few years, it is possible to get a deeper look into the previously unknown sequence of the Pakistani *N. fowleri* circular extrachromosomal ribosomal DNA (CERE - rDNA).

*N. fowleri* possesses numerous extrachromosomal elements, characterized by closed circular structures consisting of a single copy of ribosomal DNA (rDNA) and a substantial non-rDNA sequence. Despite the existence of potential open reading frames and introns, the documented transcript is solely ribosomal RNA. Notably, a solitary DNA replication origin (ori) has been identified within the non-rDNA sequence for *N. fowleri*, strongly suggesting autonomous replication of these episomes separate from the cell’s chromosomal DNA [14]. A typical *Naegleria fowleri* contains about 5000 copies of an (CERE - rDNA) [15]. Distinct from the chromosomal DNA of the organism, this distinct circular DNA is essential to determining the genetic makeup and possible virulence factors of *N. fowleri*. Researchers are intensively examining the CERE - rDNA sequence to find molecular markers and possible therapeutic targets against *N. fowleri*, with an emphasis on its usefulness as a diagnostic marker. Though research on its properties is still underway, CERE - rDNA shows potential in identifying genetic variables affecting the pathogenicity of the amoeba [16 ]. The present study aimed to identify key aspects of CERE - rDNA, which could serve as a possible marker and hence, drug target against the Pakistani *N. fowleri* isolate.

## Methodology

### Isolation and identification of ***N. fowleri***

This research on *N. fowleri* CERE sequencing utilized the DNA extracted from the same clinical isolate (AY-27) which have been used in our recently published research [12]. It was the CSF of a 28 years-old suspected PAM patient that was cultured using non-nutrient media with *Escherichia coli* ATCC29522 (Manassas, VA, USA) for 3 days. After that, the trophozoites were shifted in distilled water at 37 °C, for a duration of 30 min and observed under wet preparation. Flagellated amoeba was separated using a 0.45-micron filter, followed by washing the filter with phosphate buffer saline (PBS) solution.

During the isolation of the DNA, we used QIAprep Spin Miniprep Kit for proper isolation of plasmid DNA followed by running 1% agarose gel. The purity of DNA was determined using NanoDrop™ 2000 Spectrophotometer (Thermo Fisher Scientific) while the concentration of DNA was evaluated using Qubit 2.0 fluorometer (Thermo Fisher Scientific). Confirmation of species was done using the ITS-based PCR detection method.

gDNA quantity was optimized to 1ng for library preparation using Nextera XT DNA library preparation kit (Illumina, San Diego, CA, US). Tagmentation and adaptor-mediated amplification were carried out following the vendor’s protocol. After cleanup, the library size was evaluated using Agilent Bioanalyzer. The library was pooled and sequenced using Illumina HiSeq sequencing technology.

### Circular extrachromosomal ribosomal DNA sequencing data analysis

FastQC version v0.11.5 was used for the quality assessment of raw sequencing data. Reads having read size lower than 50 bp were removed using Sickle v 1.3 [17, 18]. Reads were mapped against reference CERE-rDNA ATCC0894 (accession no: CM017919.1), using Burrows-Wheeler Alignment BWA (BWA-MEM) tool [19]. Mapped reads were assembled using SPAdes, using the global alignment method [20]. RNA families were analyzed using the Rfam 14.2 database to annotate noncoding RNAs 18 S, 5.8 S, and 28 S rRNA gene sequences [21]. Using the ORF finder in The Sequence Manipulation Suite, ORFs were located along with their translation products. Later these translational products were confirmed using curated databases [22]. Repeats were predicted using Repeat-Modeler v1.0.11, RECON v1.05, Repeat-Scout v1.0.5, BLAST search tool against the NCBI database and against the sequenced organism itself, using Mega-BLAST [23, 24]. Aligned reads were used to call variants using SAM tools [25], CERE - rDNA variants across our isolates, with Accession no: OD958550.1, and CM017919.1 were then detected.

### Phylogenetic analysis

The phylogeny analysis was done to evaluate the evolutionary relationship among our isolated *N. fowleri* and those already sequenced and available in public databases, including: *N. fowleri* (Accession no: CM017919.1), *N. fowleri* strain LEE (Accession no: MT741533.1), *N. fowleri*Karachi_NF001 strain (Accession no: OD958550.1), *N. gruberi* (Accession no: AB298288.1), and *N. lovaniensis* (Accession no: CM010402.1). Sequences were aligned using MUSCLE and a phylogenetic tree was constructed via the maximum-likelihood method, using the MEGA-X software [26]. For further confirmation of the evolutionary relationship of various strains, we analyzed internal transcribed spacer 1 (ITS-1), 5.8 S ribosomal RNA gene for the evolutionary relationship among *N. fowleri, N. gruberi* and *N. lovaniensis.* Sequences were aligned pairwise and a phylogenetic tree was constructed using the maximum-likelihood method (with a bootstrap value of 1000 to remove biases in tree construction). As these species are closely related, recombination events were evaluated using the RDP4 software package, integrating seven algorithms (RDP, GENECONV, Chimaera, MaxChi, BootScan, SiScan, and 3Seq) [27]. A recombination event having no significant *p*-value was not considered true recombination and was eliminated from the analysis.

### Physicochemical properties and subcellular localization of circular extrachromosomal ribosomal DNA ORFs

The isolated CERE - rDNA comprised of hypothetical proteins only that were analyzed for physicochemical properties, including molecular weight, aliphatic index (AI), isoelectric point (pI) extinction coefficients, GRAVY (Grand average of hydropathy) using web server ProtParam (http://web.expasy.org/protparam/), an online tool of the ExPASY suite [28]. Subcellular localization and solubility predictions were carried out by CELLO2GO (http://harrier.nagahama-i-bio.ac.jp/sosui/) [29, 30].

### Functional prediction of hypothetical proteins

Due to a lack of structural and functional information about *Naegleria fowleri* proteins, the individual domains of CERE - rDNA hypothetical proteins were searched for their functional prediction using NCBI conserved Domain Search (CD Search), (https://www.ncbi.nlm.nih.gov/Structure/cdd/wrpsb.cgi), Pfam (https://pfam.xfam.org/), and InterProScan (http://www.ebi.ac.uk/Tools/services/web/toolform.ebi?tool=iprscan5) [31]. Using RPS-BLAST (Reverse Position Specific BLAST), conserved domains were compared in sequences present in Conservation Domain Database (CDD) using position-specific score matrices resulting from conservation domain alignment. The protein family database (Pfam) identifies proteins based on multiple sequence alignments generated using Hidden Markov Models (HMMs). Motifs from protein sequences were predicted using the online server MOTIF (http://genome.jp/tools/motif/). Proteins were searched for their homology in the NCBI database using BLASTp (http://www.ncbi.nlm.nih.gov/) against a non-redundant database with default parameters.

### Secondary structure prediction

Using online tools, secondary structures were predicted via self-optimized prediction methods including SOPMA (https://npsaprabi.ibcp.fr/cgi-bin/npsa_automat.pl?page=/NPSA/npsa_sopma.html), PSIPRED (http://bioinf.cs.ucl.ac.uk/psipred/), and ENDscript (http://endscript.ibcp.fr/ESPript/ENDscript/). For each tool, results were validated for a higher confidence rate.

### 3D model construction and druggable pocket identification

3D models were constructed for hypothetical proteins only (*n* = 4) as they showed relative query coverage and functional domains. Using the I-TASSER server (https://zhanggroup.org/I-TASSER/) [32], which functions on threading-based 3D structure prediction technique and employ multiple template structures for this purpose, the 3D models were obtained and their qualities were analyzed based on z-score [33]. Further quality evaluation of the model was done with PROCHEK, Verify3D, QMEAN, and ExPASY servers of the SWISS-MODEL workspace, along with ERRAT [34]. The model was superimposed, based on the best hit in the BLAST database (PDB ID: 3VKG), and protein pockets were analyzed using superimposition in UCSF Chimera software [35].

### Virtual screening of ZINC drug-Like compounds

Using the ligand **(**ADENOSINE-5’-DIPHOSPHATE)present in the proximity of the superimposed protein, a template site was chosen for pharmacophore-based screening of compounds and out of 12,000 compounds from a ZINC drug-like library, 1271 compounds were prioritized using MOE (Molecular Operating Environment) v2019.0102 with placement = Triangle Matcher, Rescoring 1 = London dG, refinement = forcefield, rescoring 2 = affinity dG. A classical simulation was carried out and Molecular Mechanics Poisson-Boltzmann Surface Area (MM/PBSA) energy values were then calculated for the protein. Best docked molecules were selected based on their lower energy and S-score in a particular pose. Selected compounds were further screened based on their Absorption, Distribution, Metabolism, and Excretion (ADME) using ADMESTAR 2.0 **0** (http://lmmd.ecust.edu.cn/admetsar2) and Swiss-ADME (http://www.swissadme.ch/) [36, 37]. Compounds with high blood-brain penetration and low toxicity levels were finalized.

## Result and discussion

**Fig. 1:**
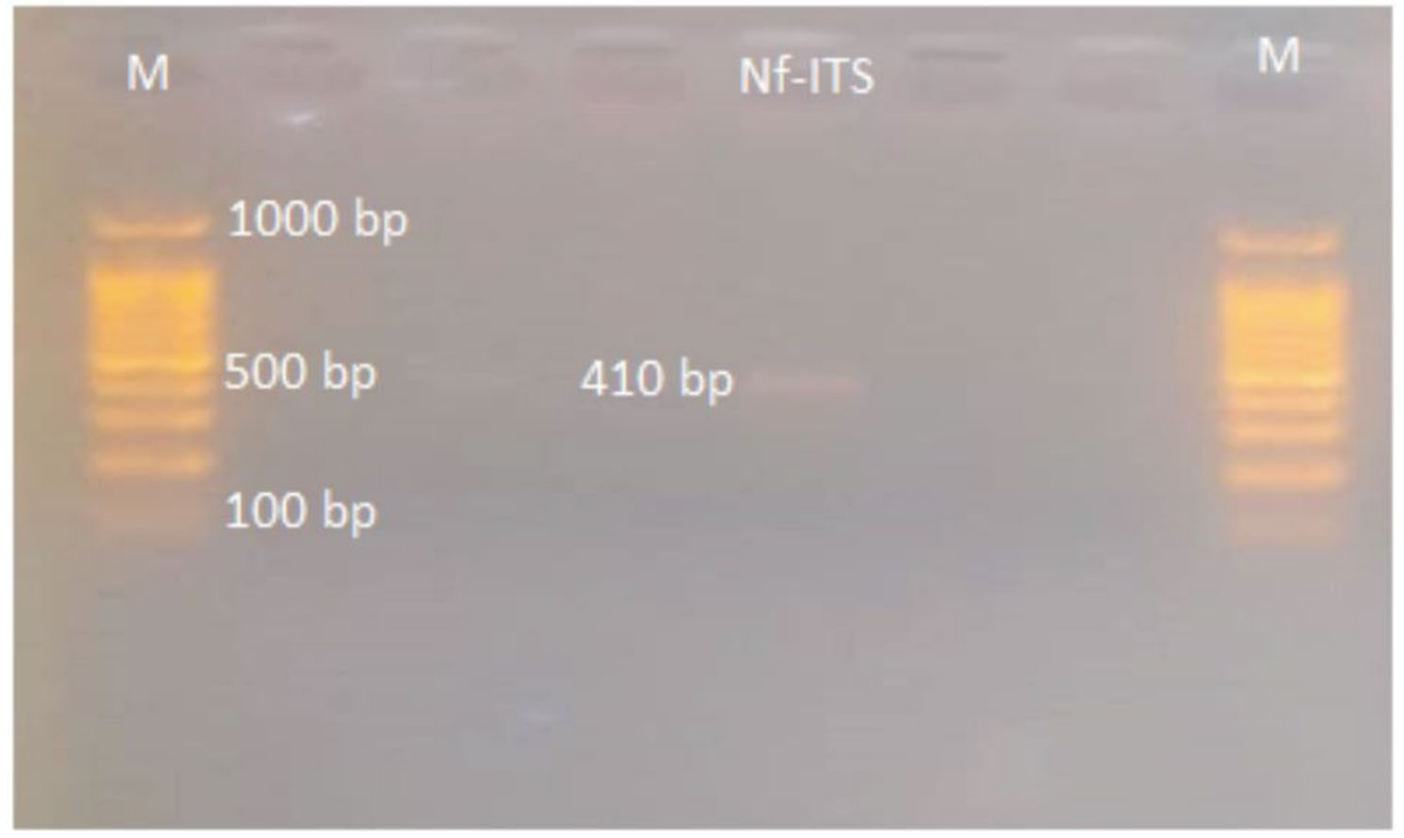
ITS region PCR based amplification for identification of *N. fowleri*

*N. fowleri* analysis under wet film observation was positive for the enflagellation test. Other morphological observations showed amoebic/cyst form, from CSF and culture. Both of these were considered for proper identification of the pathogen. Protruding pseudopodia was observed in fresh CSF wet preparation (Supplementary file 1.mp4). Further, to confirm *Naegleria fowleri*, 18 S-ITS1-5.8 S-ITS2-28 S region of 410 bp was amplified using PCR as shown in figure-1.

### ***N. fowleri*** CERE assembly and annotation

**Fig. 2:**
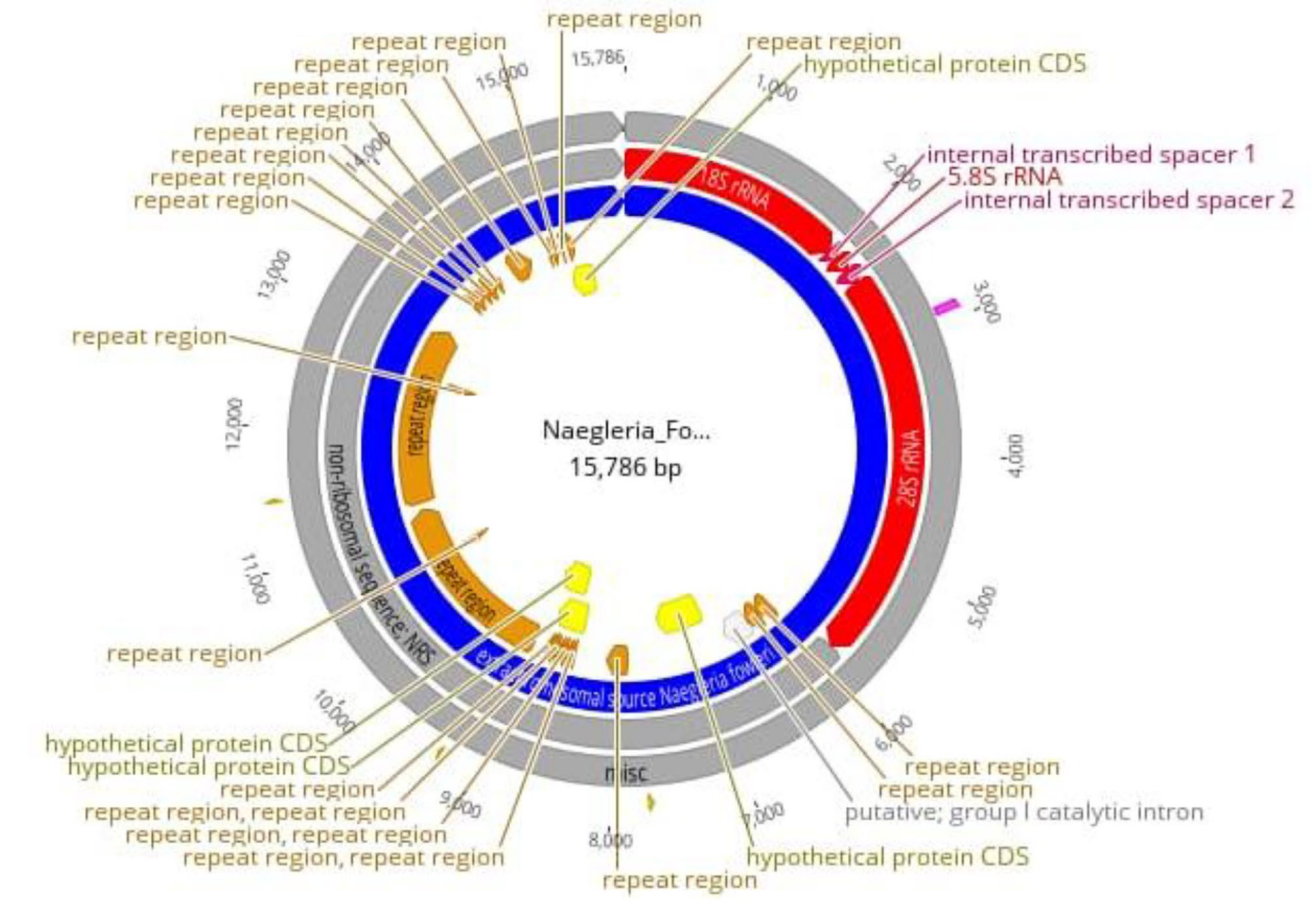
CERE - rDNA map showing various elements and their positions with repeats and hypothetical proteins

CERE-rDNA sequence data was quality-filtered for further downstream processing. High-quality assembly reads were first aligned against *N. fowleri* (CM017919.1) followed by the assembly of the CERE - rDNA genome. The major features of CERE elements include rRNA genes, repeats, and ORFs. The CERE-rDNA size of *N. fowleri* Karachi isolate that we sequenced is up to 15.79 kb having 40.5% GC content **(**Figs. 1 and 2**). However**, the previously identified CERE-rDNA from different Nagleria spp shows some variation in its size; The CERE-rDNA size is 15.79 kb in *N. fowleri* strain LEE MT741533.1, 15 kb in *N. gruberi*, 14 kb in *N. lovaniensis*, 15 kb in *N. jamiesoni*, 13.6 kb in *N. australiensis* and 11.8 kb in *N. jadini* [3–6]. Most of the size difference in CERE elements of different Naegleria specie is due to the variation in their non-rDNA sequence (NRS); however, their rDNA sequence have almost similar size having only minor differences in the internal transcribed spacers (ITS) [16].

**The** 18 S in CERE element comprised of 2027 bp followed by two internal repeats of 223 bp separated by 144 bp 5.8 S. The 28 S rRNA comprised 3465 bp followed b y repeat elements and hypothetical proteins. Repeat elements comprised 7268 bp (46.04) in the whole CERE element.

Our *N. fowleri* isolate was compared with other *N. fowleri* isolates to assess their evolutionary relationship and single nucleotide polymorphisms (SNPs), along with insertions and deletions. Variants with a quality score of less than 30 were removed. Variants showed higher variability among various *N. fowleri* isolates analyzed in this study. There were 90 variants in total, including 41 variants in 18s rRNA and 49 variants in 28s rRNA region of CERE - rDNA. *N. fowleri* strain LEE MT741533.1 showed a deletion of 44 nucleotides at the 2026 position of 18s rRNA, and T to A transition on direct repeat region at position 8040. *N. fowleri* CM017919.1 had an insertion in tandem repeat at position 6,207 of about 167 nucleotides. The second insertion was of 22 nucleotides as a direct repeat at position 14,982. A deletion (direct repeat from ACCC to ACC at position 12,354) was also seen. Besides these insertions and deletions, 11 SNPs were also present (Supplementary file 2).

### Phylogenetics and recombination events

**Fig. 3:**
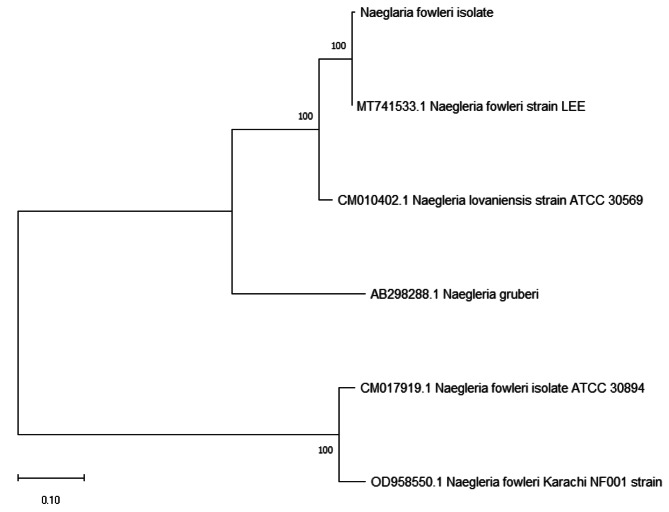
Phylogenetic tree showing relatedness of the present CERE - rDNA isolate with other CERE - rDNA

**Fig. 4:**
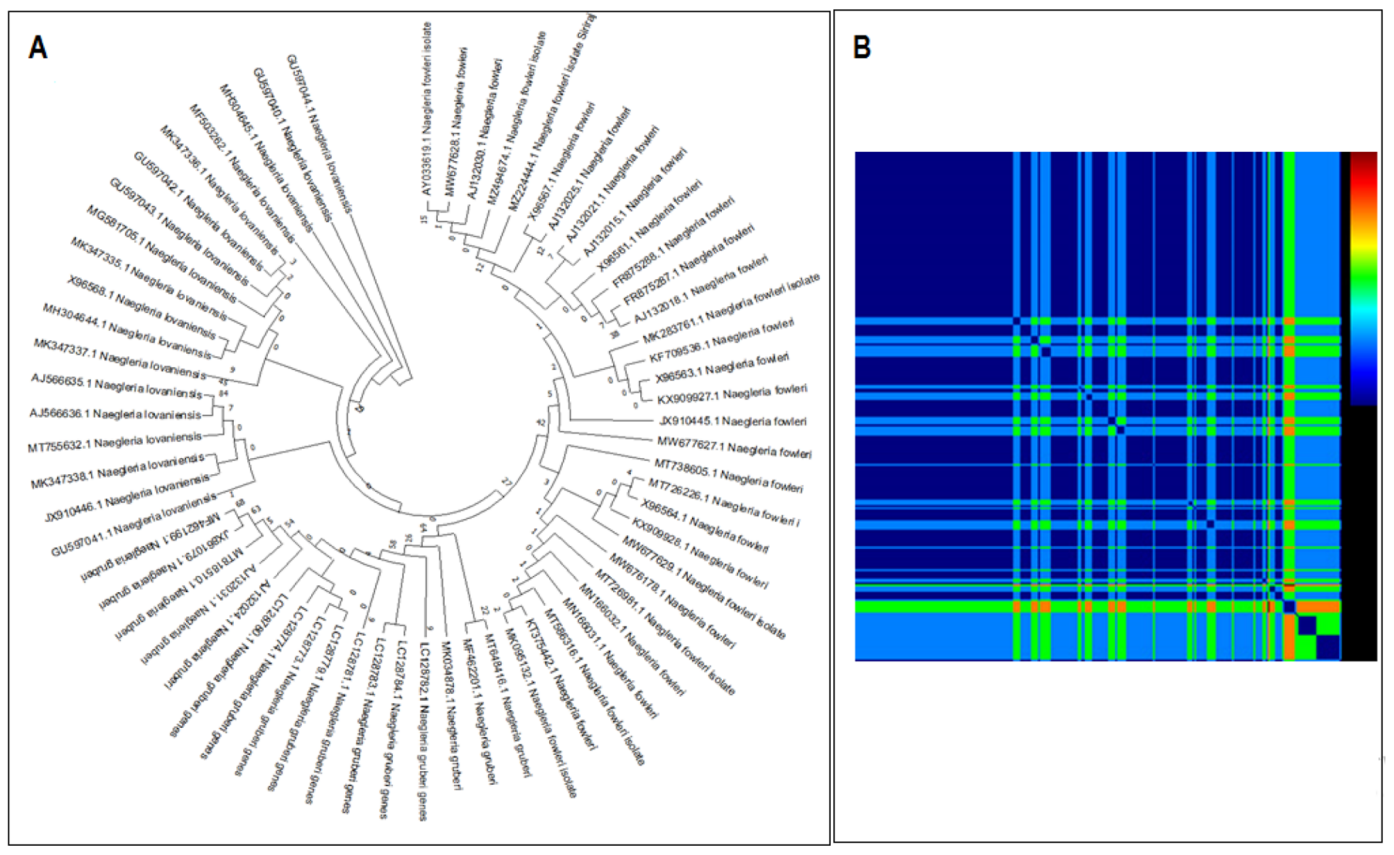
**(A)** ITS-1 based phylogenetics analysis for proper classification of various *Naegleria* isolates. **(B)** Recombinational events map showing various recombinational events between various different *Naegleria* types

Our isolate and *N. fowleri* (CM017919.1), *N. fowleri* strain LEE (MT741533.1), *N. fowleri* Karachi_NF001 strain (OD958550.1), *N.gruberi* (AB298288.1), and *N. lovaniensis* (CM010402.1) were studied using Neighbor-Joining method to evaluate evolutionary relationship across various CERE - rDNA. The ITS-I DNA sequences from all CERE- rDNA of different species included here for phylogenetic analyses showed different patterns of evolutionary relationship (Fig. 3**).** CERE-rDNA DNA sequences of *N. fowleri* Karachi isolate showed maximum similarity with *N. fowleri* strain LEE and *N. lovaniensis* CERE-rDNA sequence also showed close homology. *N. gruberi* formed a separate group, while *N. fowleri* Karachi_NF001 and *N. fowleri* species(CM017919.1) were forming a separate clade. This observation is quite interesting because all CERE-rDNA sequences used for phylogeny analyses belong to separate species. Hence a regularly used internal transcribed spacer I and 5.8 S ribosomal RNA genes were considered for further phylogenetic analysis. The phylogenetic tree was constructed using 66 sequences. It showed that the pattern of evolution and clade formation was different for different species (Fig. 4A**)**. These analyses indicate that ITS-I, ITS-II, and 5.8 S rRNA are of great diagnostic value for rapid amoeboid identification and differentiation. The Karachi isolate CERE - rDNA showed a different pattern in the NJ tree, this could be due to low sample size as a low number of CERE - rDNA have been reported so far. A total of 22 recombination events were predicted and these were screened for actual recombination events (Fig. 4B**)**. The analyses resulted in some over-expressed sequences and were subsequently eliminated. Further stringency was increased by considering parent recombination events (from both major and minor parental recombinations), identified by their presence in both sequences. Among 5 recombination events, three were found among *N.gruberi* (AB298288.1) and *N. fowleri* strain LEE (MT741533.1) CERE - rDNA, starting from 9,366 to 9,754 bp,13,485 to 13,772 bp, and 11,892 to 11,994 bp, respectively. *N.gruberi* (AB298288.1) and OD958550.1 showed two recombination sites (23,461–24,449) while other recombination events occurred between *N.gruberi* (AB298288.1) and *N. fowleri* strain LEE (MT741533.1) (22,649–23,287) (Supplementary file 3). These recombination events could explain possible variability among the different patterns in terms of genetic heterogeneity (Fig. 3**)**.

### Hypothetical protein structures and functionality evaluation

Four hypothetical proteins were studied for their physiological and biochemical analysis. The hypothetical protein 4 (Hypo-4) containing 104 amino acids, showed a molecular weight of.

10552.20 Dalton, theoretical pI: 11.63, and Grand Average of Hydropathicity (GRAVY): 0.284. Hypo-4 protein was classified as stable with an estimated half-life of 20 h (> 20 h in yeast having the instability index (II) computed to be 11.15) [38].

### Protein structure and model quality assessment

**Fig. 5:**
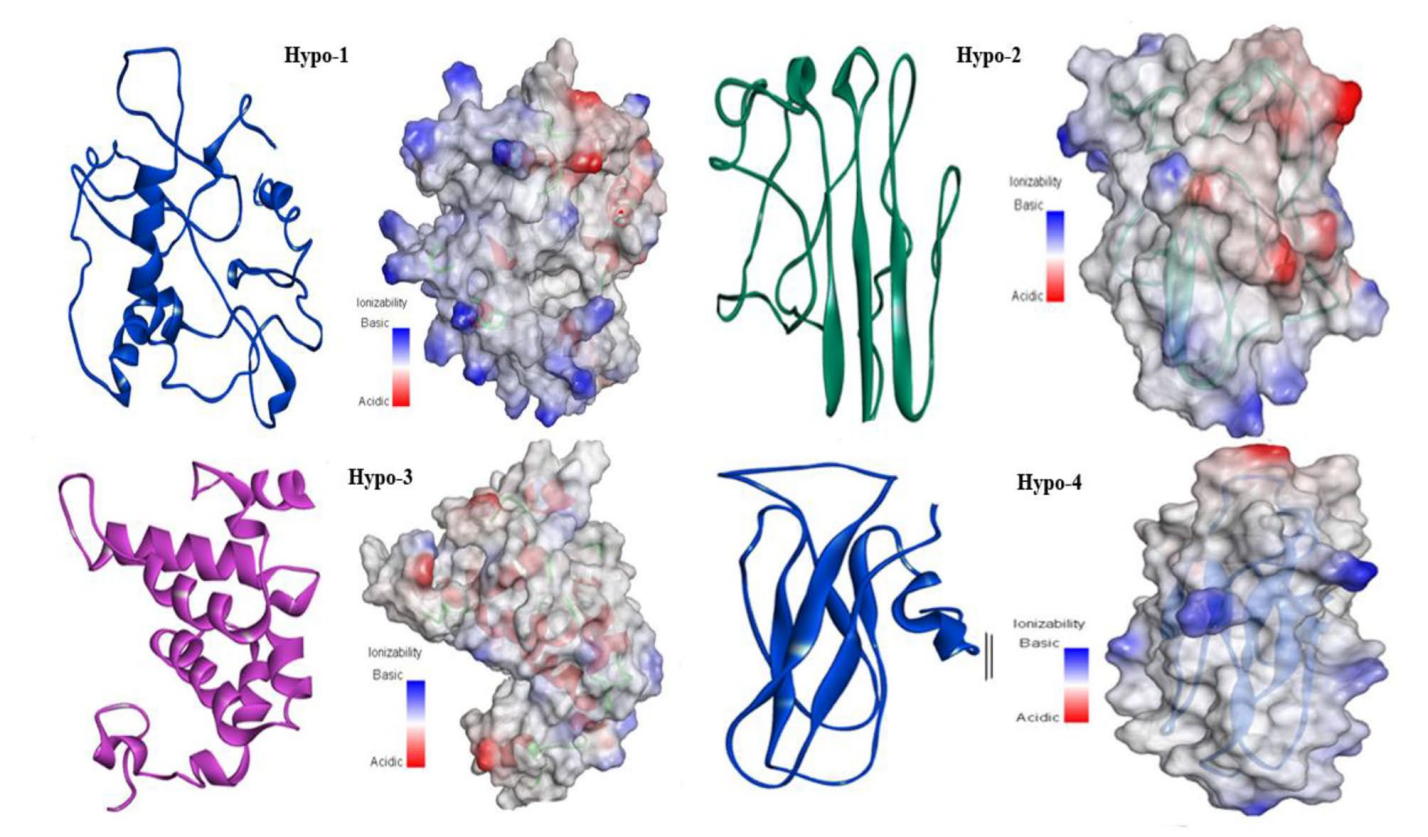
Hypothetical proteins presented in ribbon form and surface view showing different orientations of α-helix and β-sheets

All hypothetical proteins including Hypo-1, Hypo-2, Hypo-3, and Hypo-4 were studied for their proper secondary structures using PSIPRED, SOPMA, and ENDscript servers. In three hypothetical proteins, the random coil was the most predominant feature with 53.85%, 49.30%, and 60.58% occurrence in Hypo-1, Hypo-2, and Hypo-4, respectively, while in Hypo-3, it was only 20.55%. Hypo-2 and hypo-4 proteins belong to all b-class of protein folds and are found to consist of 12.68% and 13.46% b-elements, respectively. ENDscript and PSIPRED showed similar results. The Models were predicted by I-TASSER and checked for proper structure prediction using I-TASSER scoring. The structure for Hypo-1 proteins lacks regular secondary structure, although its fold has few a-helices. The majority of the surface area showed basic potential, localized on one side of the protein which may be involved in nucleotide binding. This protein was predicted to have ATP binding/ligase activity. The Hypo-2 protein (Fig. 5) had 50% loop region while the other 50% consisted of b-sheets having three b-strands, representing the mixed type of surface potential. It was predicted to have ribonuclease-inhibitor activity. The Hypo-3 protein is included in all a-class of proteins. It has ATP binding/glucosidase activity. The Hypo-4 protein belongs to all b-class of proteins and is predicted to contain hydrolase activity against the O-glycosyl compounds e.g., polysaccharides. The surface representation of this protein depicts the overall basic surface potential which facilitates the binding of this protein with polysaccharide.

### Protein binding site and virtual screening

**Fig. 6:**
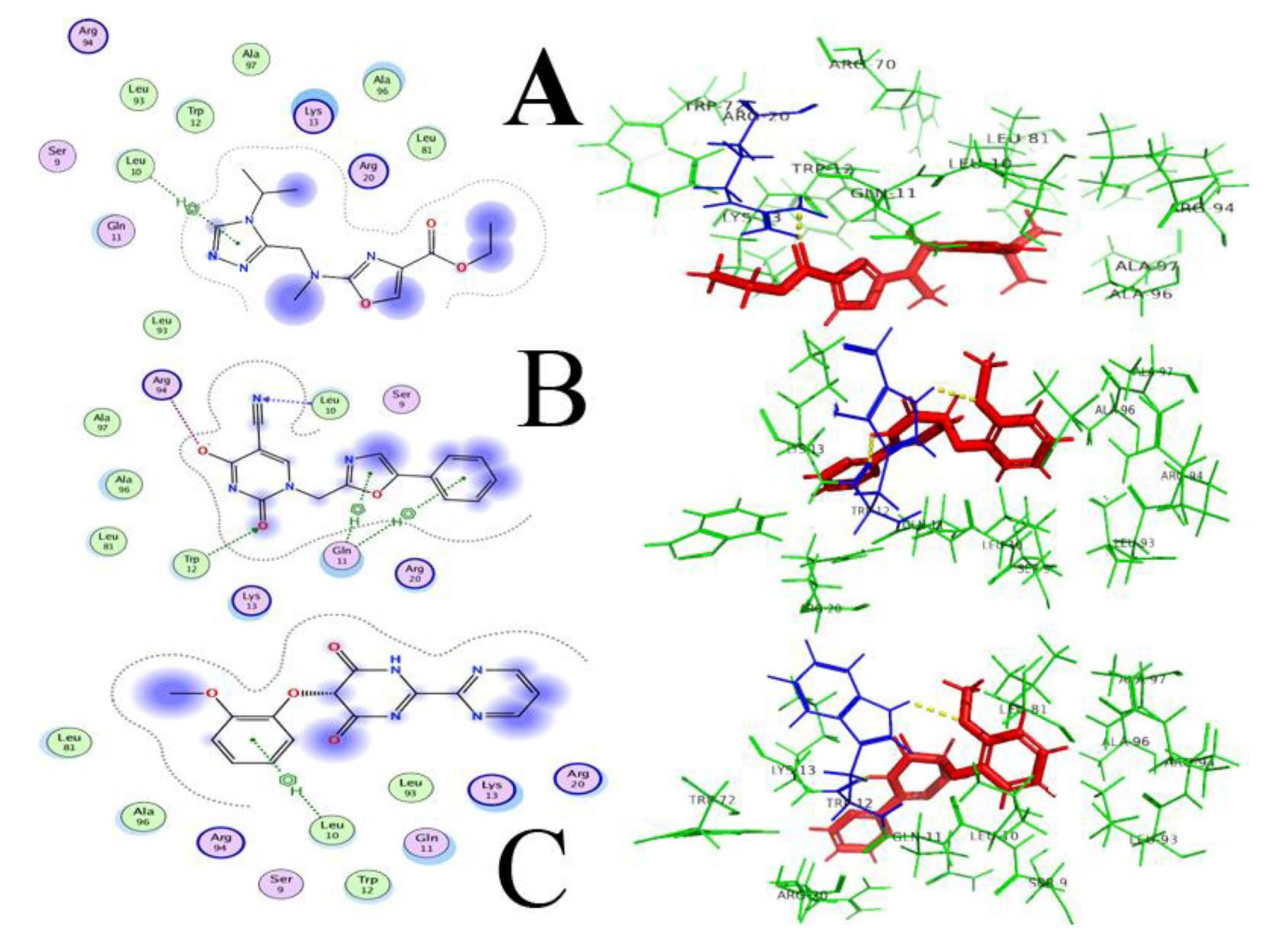
ZINC77564275, ZINC48229542 and ZINC15022129 are shown in figure **A**, **B** and **C** respectively. Polar and non-polar residues are shown in dark blue and green color, respectively

As Hypo-4 depicted a proper conformation and structure, it was selected for further analysis. Domain conservation analysis along with functional annotation and functional site identification was performed using BLASTp search, NCBI-CD Search, Pfam, and InterProScan. The motor domain of *Dictyosteliumdiscoideum* cytoplasmic dynein (PDB ID: 3VKG) was selected as a template that showed similarity to hypo-4 protein, having 15% query coverage and 68.75% sequence identity with an E-value of 0.020. The structural superimposition was performed to check the structural similarity and active site comparison in both structures, where adenosine-5’-diphosphate ligand was bound nearby of the template structure. This ligand structure was used for pharmacophore-based screening (11 features selected in MOE software), with a ZINC library of 11,193 drug-like molecules. 1,271 compounds gave the best hits in virtual screening and the top 20 hits (Supplementary file 4) were tabulated considering their S-value and were further subjected to ADME analyses (Administration, Distribution, Metabolism, and Excretion) and toxicity. Compounds with ID: ZINC77564275, ZINC48229542, and ZINC15022129 were selected as potential drugs based on their S-score and interaction parameters (Fig. 6). These top compounds having higher binding affinities were then analyzed for pharmacokinetics and pharmacodynamics using their administrant, distributions, metabolism, and excretion (ADME) profile. Compounds showing blood-brain permeability should be key for considering pathogen that resides in the brain for its pathogenesis, as drug molecules need to cross blood blood-brain barrier. All compounds were predicted substrates of P-glycoprotein, showing no inhibitory effect against the cytochromes CYP3A4, CYP2C9, CYP2C19, CYP2D6, and CYP1A2. No compound depicted potential carcinogenicity while ZINC48229542 was positive for Ames mutagenesis, and probably not be a suitable drug.

ZINC77564275 (ethyl 2-(((4-isopropyl-4 H-1,2,4-triazol-3-yl)methyl)(methyl)amino)oxazole-4-carboxylate) and ZINC15022129 (5-(2-methoxyphenoxy)-[2,2’-bipyrimidine]-4,6(1 H,5 H)-dione) were finalized, based on ADME toxicity analysis. These two compounds showed least to no toxicity to the host and honey bees, crustacea, and fish. Selected final compounds were also positive for biological degradation, and safe for the environment. To the best of our knowledge, the proposed compounds are novel and safe, as they have not been previously reported in the literature for their anti-Naegleria-like activities.

## Conclusion

The large-sized sequenced CERE of the *Naegleria fowleri* species is of interest as it encodes the organisms’ ribosomal RNA. At present, our understanding of CERE biology is derived from complete CERE sequences, so it is important to acquire more CERE sequences from other *Naegleria fowleri* species. We identified and theoretically explored the existence of circular extrachromosomal rDNA elements in the CSF PAM Patient for the first time. The CERE covers 15,786 bp and consists of a single copy of the organism’s rDNA cistron. The non-ribosomal sequence contains four potential open reading frames, two large direct repeat sequences, and numerous smaller repeated-sequence regions. The ORFs (open reading frames) were modeled and targeted with a library of – 12,000 drug-like compounds, that were initially retrieved from the ZINC database. The model-template superimposition highlighted the core druggable site/s and their active site residues of the targeted proteins employed for pharmacophore-based (lead-based) drug designing. Two putative ZINC compounds were finally selected after docking analyses based on various physicochemical properties and it is proposed that these ZINC compounds could serve as potential inhibitors for CERE-based *N. fowleri* hypothetical proteins. Here, we report for the first time, the CERE - rDNA genome sequence of a fatal amoeba isolated from a CSF patient in Karachi, Pakistan, but also, we have characterized their hypothetical proteins through their 3D structures as well as screened inhibitors that require future laboratory validations to overcome the onsets of *N. fowleri*.

### Electronic supplementary material

Below is the link to the electronic supplementary material.

Supplementary Material 1

Supplementary Material 2

Supplementary Material 3

Supplementary Material 4

Supplementary Material 5

Supplementary Material 6

## Data Availability

The data that support the findings of this study are openly available in NCBI at [https://www.ncbi.nlm.nih.gov/search/all/?term=MZ430524], Accession no. MZ430524.1.
