# Correction: Exploring the extrachromosomal plasmid rDNA of Naegleria fowleri AY27 genotype II: a human brain-eating amoeba via highthroughput sequencing

**DOI:** 10.1186/s12920-024-01912-9

**Published:** 2024-05-23

**Authors:** Muhammad Aurongzeb, Hafiz Muhammad Talha Malik, Muhammad Jahanzaib, Syed Shah Hassan, Yasmeen Rashid, Tariq Aziz, Metab Alharbi

**Affiliations:** 1 Department of Biotechnology, Faculty of Engineering Sciences & Technology, Hamdard University, Karachi, 74600 Pakistan; 2 Alpha Genomics Private limited, Islamabad, Pakistan; 3 International Center for Chemical and Biological Sciences, JRC Genome Research, PCMD, University of Karachi, Karachi, 75270 Pakistan; 4 Department of Biochemistry, University of Karachi, Karachi, 75270 Pakistan; 5 Laboratory of Animal Health Food Hygiene and Quality, Department of Agriculture, University of Ioannina, Arta, 47132 Greece; 6 Department of Pharmacology and Toxicology College of Pharmacy, King Saud University, Riyadh, Saudi Arabia

Correction to: Aurongzeb et al. BMC Medical Genomics (2024) 17:125

10.1186/s12920-024-01890-y

Following the publication of the Original Article, the authors reported errors as follows:

The name of the second author was written incorrectly as Muhammad Talha Hafiz Malik. The correct name is Hafiz Muhammad Talha Malik.

Furthermore, in the Original Article, Muhammad Jahanzaib and Syed Shah Hassan were wrongly affiliated to the Department of Biotechnology, Faculty of Engineering Sciences & Technology, Hamdard University, Karachi 74,600, Pakistan. Syed Shah Hassan was also mistakenly affiliated to the Centre for Technological Development in Health (CDTS), Oswaldo Cruz Foundation (Fiocruz), Av. Brasil 4036 - Manguinhos, Rio de Janeiro, RJ 21040-361, Brazil. The correct affiliation for both authors is JRC Genome Research, PCMD, International Center for Chemical and Biological Sciences, University of Karachi, Karachi 75,270, Pakistan. The authors’ names and affiliations are presented correctly in this article.

Lastly, the first sentence of the “Ethics approval and consent to participate” mentioned “Not applicable”. This is not relevant, thus has been removed.

The original article has been corrected.

